# Supplementary material for: Cancer Cell’s Achilles Heels: Considerations for Design of Anti-Cancer Drug Combinations
Source: Int J Mol Sci. 2024 Dec 17;25(24):13495. doi: 10.3390/ijms252413495 (PMC11676151; doi:10.3390/ijms252413495)
Supplement: Supplementary file 1 [file ijms-25-13495-s001.zip › ijms-3377637-supplementary/Supplementary_Files/SUPPLEMENTARY FIGURES AND TABLES.pdf]

SUPPLEMENTARY FIGURES AND TABLES

Cancer Cell's Seven Achilles Heels: Considerations for design of anti-cancer drug combinations.

Valid Gahramanov[1,2], Frederick S. Vizeacoumar[3], Alain Morejon Morales[3,4], Keith Bonham[5], Meena K. Sakharkar[6], Santosh Kumar[2], Franco J. Vizeacoumar[5], Andrew Freywald[3], Michael Y. Sherman[2*]

1. Molecular, Cellular, and Developmental Biology, Yale University, CT06511, USA
2. Department of Molecular Biology, Ariel University, Ariel 40700, Israel
3. Department of Pathology and Laboratory Medicine, College of Medicine, University of Saskatchewan, Room 2841, Royal University Hospital, 103 Hospital Drive, Saskatoon, SK, S7N 0W8, Canada.
4. Department of Biochemistry, Microbiology and Immunology, University of Saskatchewan, GA20 Health Sciences, 107 Wiggins Road, Saskatoon, SK, S7N 5E5, Canada.
5. Cancer Research, Saskatchewan Cancer Agency and Division of Oncology, University of Saskatchewan, 4D30.2 Health Sciences Building, 107 Wiggins Road, Saskatoon, SK, S7N 5E5, Canada.
6. Drug Discovery and Development Research Group, College of Pharmacy and Nutrition, University of Saskatchewan, 107 Wiggins Road, Saskatoon, SK S7N 5E5, Canada

*. To whom correspondence should be sent

Corresponding author: Michael Y. Sherman - sherma1@ariel.ac.il

Table S2: Identification of common signaling pathways among the shRNA screening data. The tables below represent the pathways that were found in at least 2, 3, 4, and 5 independent screens.

Pathways are present at least in 2 shRNA screens.
KEGG_TGF_BETA_SIGNALING_PATHWAY
KEGG_PROPANOATE_METABOLISM
KEGG_PROTEIN_EXPORT
KEGG_TOLL_LIKE_RECEPTOR_SIGNALING_PATHWAY
KEGG_GLYCOSYLPHOSPHATIDYLINOSITOL_GPI_ANCHOR_BIOSYNTHESIS
KEGG_EPITHELIAL_CELL_SIGNALING_IN_HELICOBACTER_PYLORI_INFECTION
KEGG_LEISHMANIA_INFECTION
KEGG_PYRUVATE_METABOLISM
KEGG_TYPE_II_DIABETES_MELLITUS
KEGG_INSULIN_SIGNALING_PATHWAY
KEGG_NEUROTROPHIN_SIGNALING_PATHWAY
KEGG_PENTOSE_AND_GLUCURONATE_INTERCONVERSIONS
KEGG_DNA_REPLICATION
KEGG_HOMOLOGOUS_RECOMBINATION
KEGG_PYRIMIDINE_METABOLISM
KEGG_PHENYLALANINE_METABOLISM
KEGG_OXIDATIVE_PHOSPHORYLATION
KEGG_HUNTINGTONS_DISEASE
KEGG_ECM_RECEPTOR_INTERACTION
KEGG_MAPK_SIGNALING_PATHWAY
KEGG_ARGININE_AND_PROLINE_METABOLISM
KEGG_CYTOKINE_CYTOKINE_RECEPTOR_INTERACTION
KEGG_ABC_TRANSPORTERS
KEGG_NOD_LIKE_RECEPTOR_SIGNALING_PATHWAY

Pathways are present at least in 3 shRNA screens.
KEGG_RIBOSOME
KEGG_GAP_JUNCTION
KEGG_TIGHT_JUNCTION
KEGG_PROTEASOME
KEGG_SPLICEOSOME
KEGG_CELL_CYCLE
KEGG_VEGF_SIGNALING_PATHWAY
KEGG_T_CELL_RECEPTOR_SIGNALING_PATHWAY
KEGG_UBIQUITIN_MEDIATED_PROTEOLYSIS
KEGG_ANTIGEN_PROCESSING_AND_PRESENTATION
KEGG_BETA_ALANINE_METABOLISM
KEGG_ALANINE_ASPARTATE_AND_GLUTAMATE_METABOLISM
KEGG_ENDOCYTOSIS
KEGG_CYTOSOLIC_DNA_SENSING_PATHWAY
KEGG_PATHOGENIC_ESCHERICHIA_COLI_INFECTION
KEGG_RNA_POLYMERASE
KEGG_STARCH_AND_SUCROSE_METABOLISM
KEGG_MTOR_SIGNALING_PATHWAY
KEGG_MISMATCH_REPAIR
KEGG_NUCLEOTIDE_EXCISION_REPAIR

Pathways are present at least in 4 shRNA screens.
KEGG_RIBOSOME
KEGG_GAP_JUNCTION
KEGG_TIGHT_JUNCTION
KEGG_PROTEASOME
KEGG_SPLICEOSOME
KEGG_VEGF_SIGNALING_PATHWAY
KEGG_ANTIGEN_PROCESSING_AND_PRESENTATION
KEGG_CYTOSOLIC_DNA_SENSING_PATHWAY
KEGG_PATHOGENIC_ESCHERICHIA_COLI_INFECTION
KEGG_STARCH_AND_SUCROSE_METABOLISM
KEGG_MTOR_SIGNALING_PATHWAY

Pathways are present at least in 5 shRNA screens.
KEGG_RIBOSOME
KEGG_RIBOSOME
KEGG_GAP_JUNCTION
KEGG_TIGHT_JUNCTION
KEGG_PROTEASOME
KEGG_SPLICEOSOME
KEGG_CYTOSOLIC_DNA_SENSING_PATHWAY
KEGG_PATHOGENIC_ESCHERICHIA_COLI_INFECTION
KEGG_STARCH_AND_SUCROSE_METABOLISM

Figure S1: Global view of shRNA screening data analysis. Represents the linear regression of 8 shRNA screening library correlation with the found hits. There was a linear correlation between the number of hits and the size of the library.

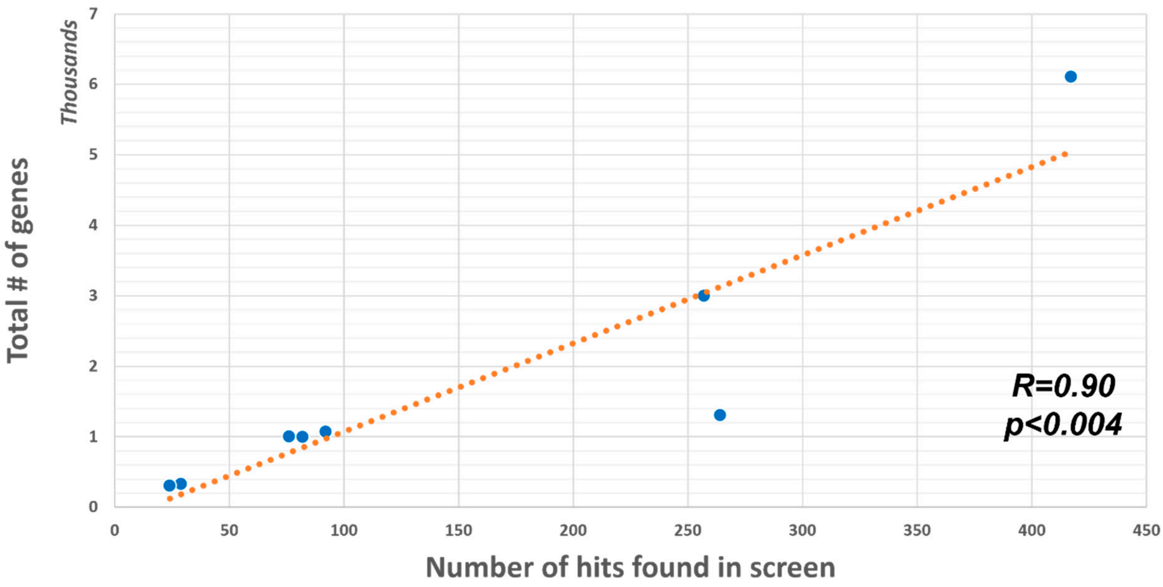

Figure S2: MDS plots demonstrate the diversity of drugs that synergize with the “weak point” pathways inhibitors roscovitine, abameciclib, and rapamycin, and “control therapeutics” cisplatin, 5-FU, and erlotinib. The area covered by drug effects-representing triangles reflects the diversity of the drugs’ actions.

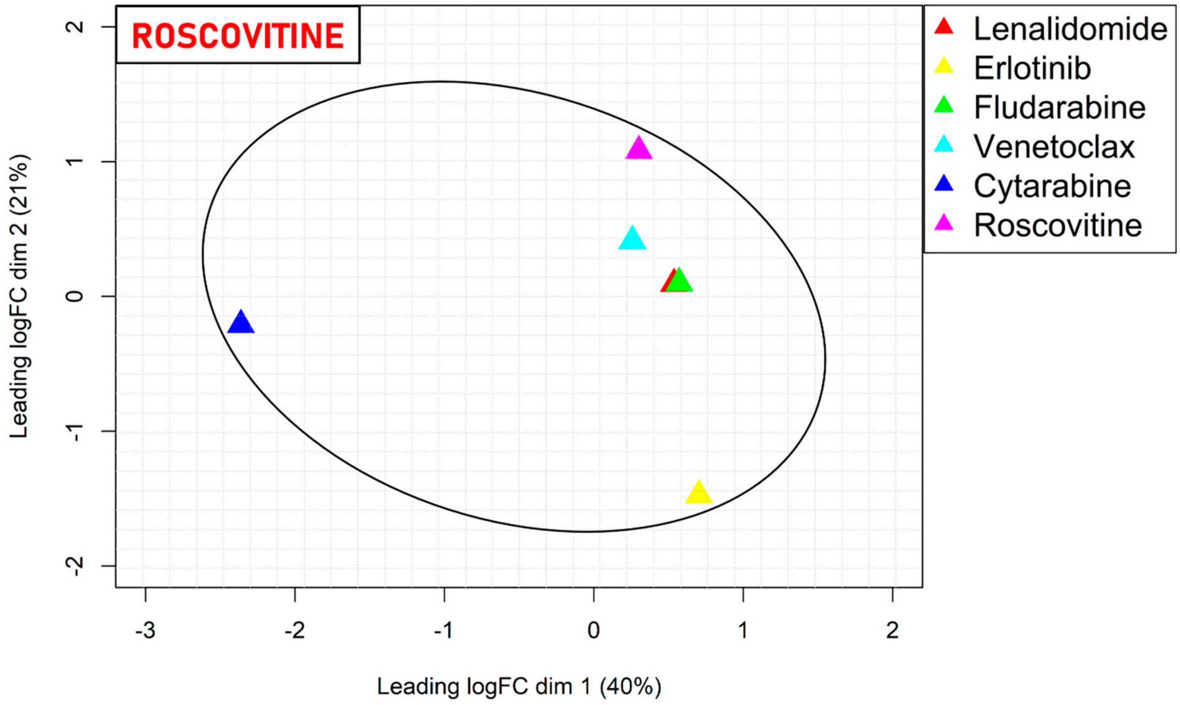

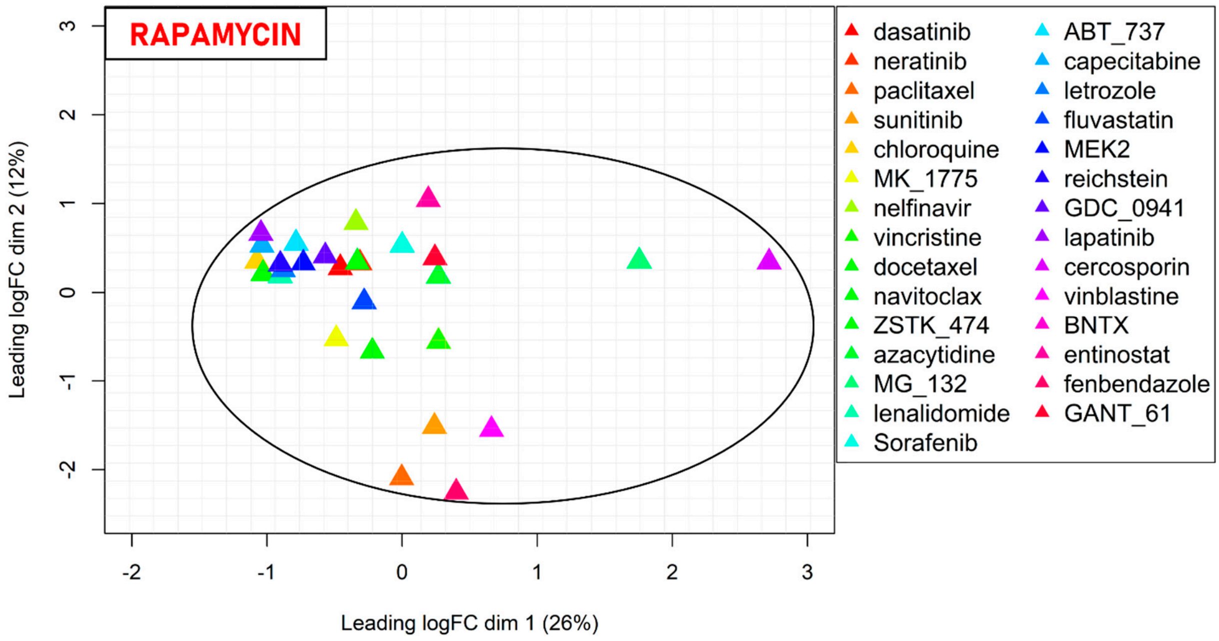

RAPAMYCIN
Leading logFC dim 2 (12%)
Leading logFC dim 1 (26%)
-2
-1
0
1
2
3
dasatinib
neratinib
paclitaxel
sunitinib
chloroquine
MK_1775
nelfinavir
vincristine
docetaxel
navitoclax
ZSTK_474
azacytidine
MG_132
lenalidomide
Sorafenib
ABT_737
capecitabine
letrozole
fluvastatin
MEK2
reichstein
GDC_0941
lapatinib
cercosporin
vinblastine
BNTX
entinostat
fenbendazole
GANT_61

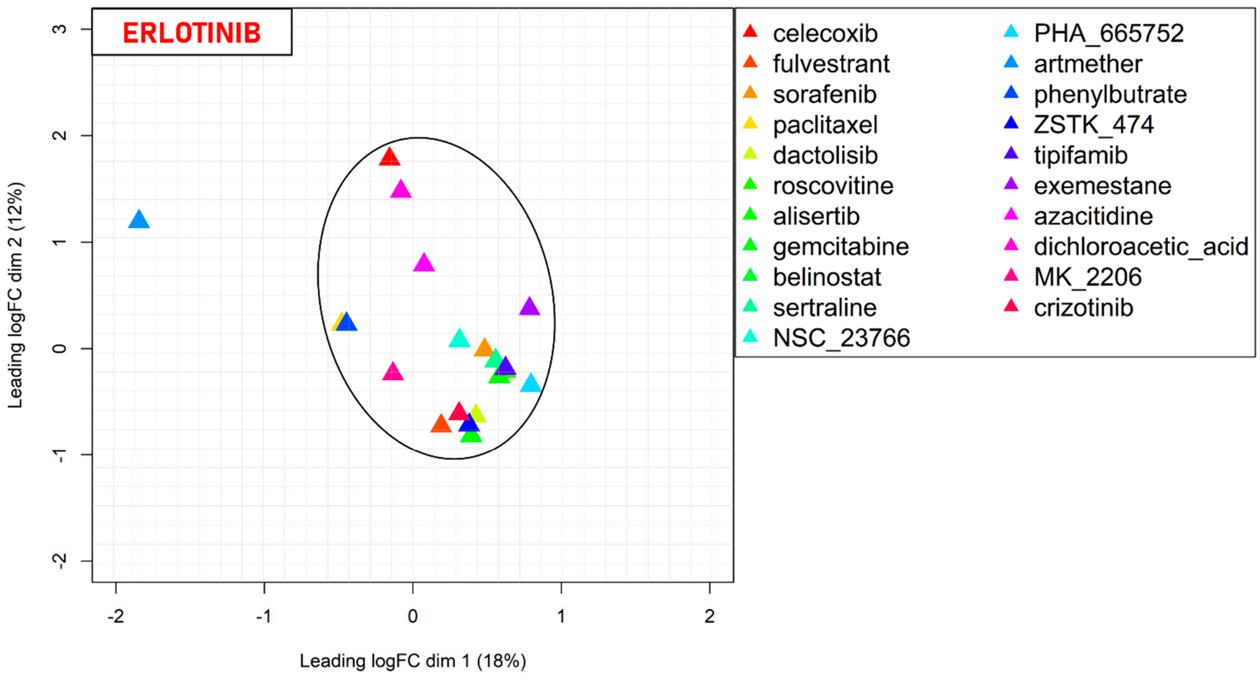
ERLOTINIB
Leading logFC dim 1 (18%)
Leading logFC dim 2 (12%)
-2
-1
0
1
2
3
celecoxib
fulvestrant
sorafenib
paclitaxel
dactolisib
roscovitine
alisertib
gemcitabine
belinostat
sertraline
NSC_23766
PHA_665752
artmether
phenylbutrate
ZSTK_474
tipifamib
exemestane
azacitidine
dichloroacetic_acid
MK_2206
crizotinib

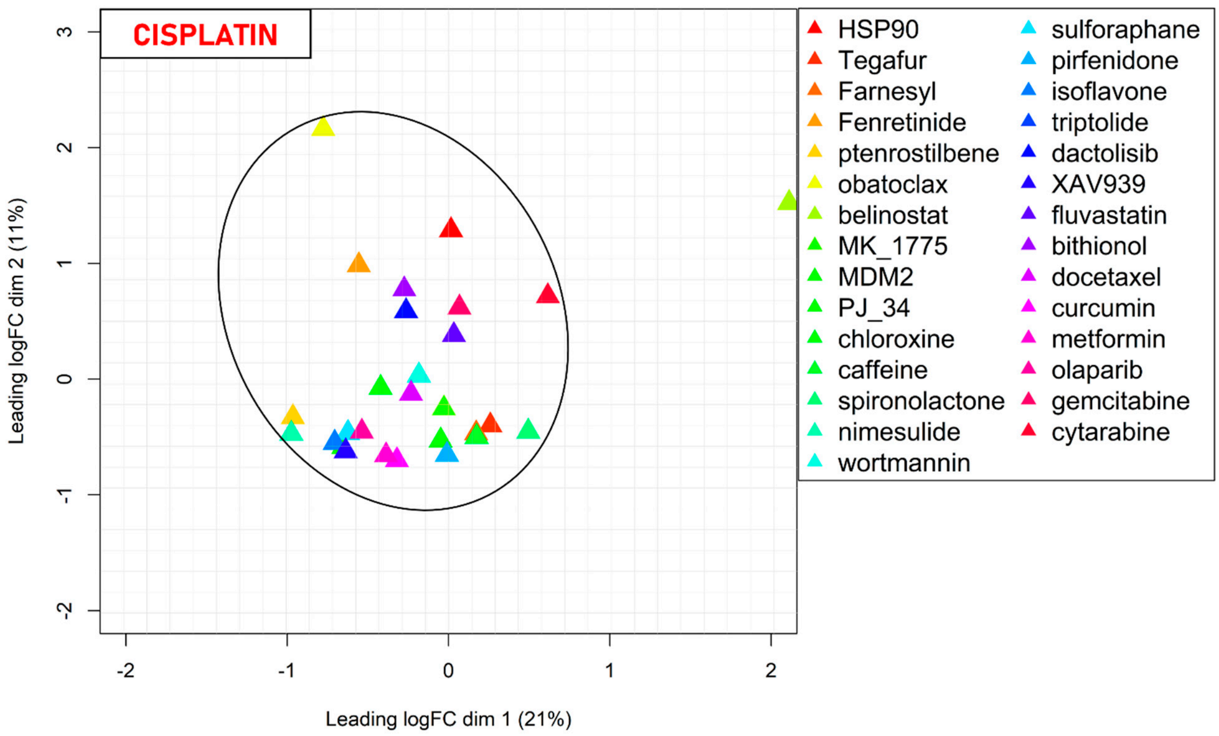
CISPLATIN
Leading logFC dim 1 (21%)
Leading logFC dim 2 (11%)
-2
-1
0
1
2
3
HSP90
Tegafur
Farnesyl
Fenretinide
ptenrostilbene
obatoclax
belinostat
MK_1775
MDM2
PJ_34
chloroxine
caffeine
spironolactone
nimesulide
wortmannin
sulforaphane
pirfenidone
isoflavone
triptolide
dactolisib
XAV939
fluvastatin
bithionol
docetaxel
curcumin
metformin
olaparib
gemcitabine
cytarabine

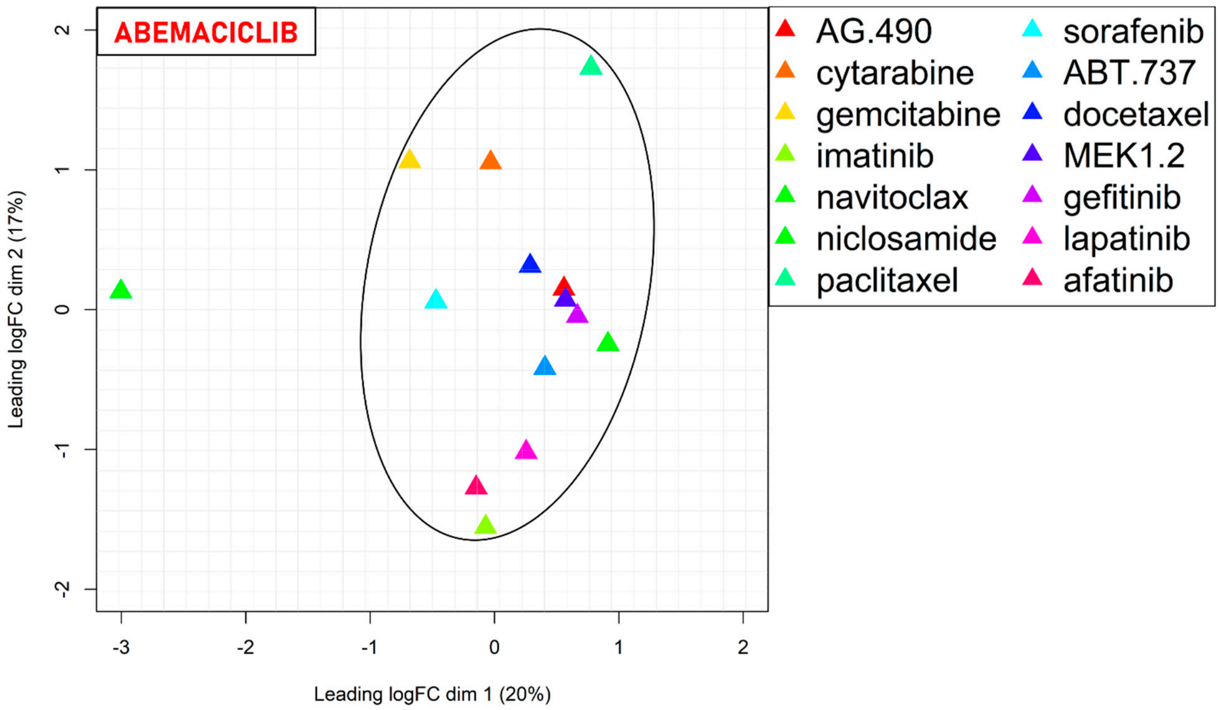
ABEMACICLIB
AG.490
cytarabine
gemcitabine
imatinib
navitoclax
niclosamide
paclitaxel
sorafenib
ABT.737
docetaxel
MEK1.2
gefitinib
lapatinib
afatinib
Leading logFC dim 2 (17%)
Leading logFC dim 1 (20%)

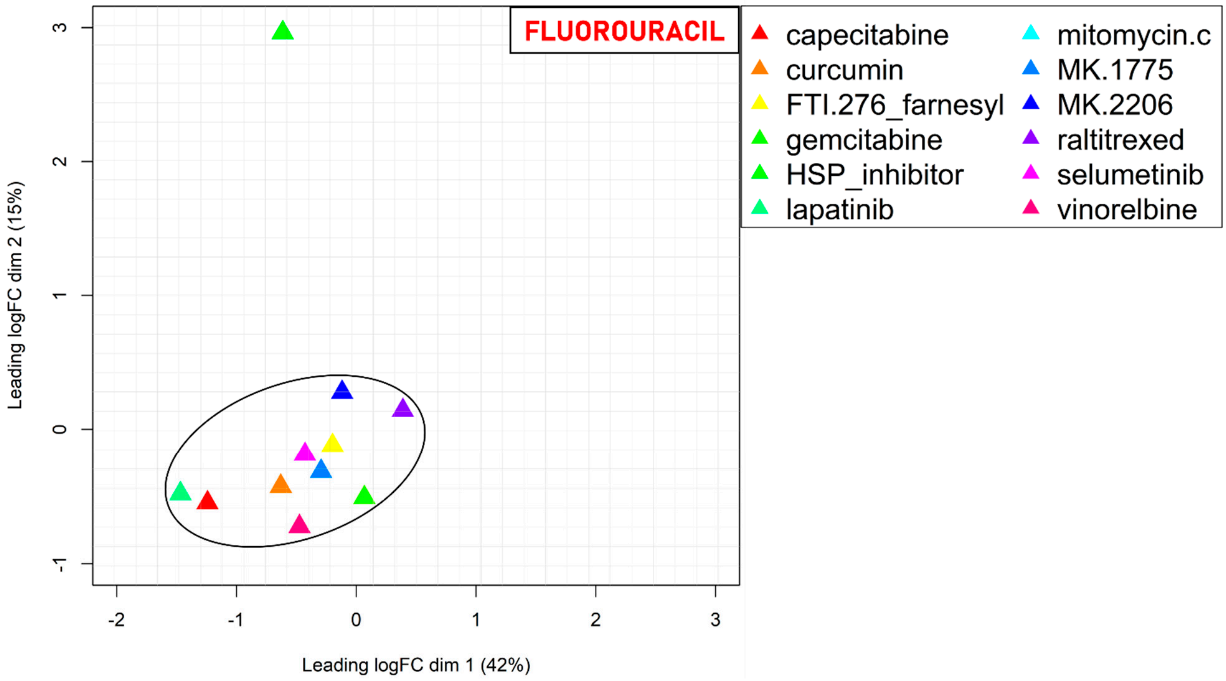
FLUOROURACIL
capecitabine
curcumin
FTI.276_farnesyl
gemcitabine
HSP_inhibitor
lapatinib
mitomycin.c
MK.1775
MK.2206
raltitrexed
selumetinib
vinorelbine
Leading logFC dim 2 (15%)
Leading logFC dim 1 (42%)
